# KDM3A knockdown regulates COMP, LOX, COL8A1 and ACOT1 genes in myocardial fibrosis

**DOI:** 10.6026/973206300200305

**Published:** 2024-04-30

**Authors:** Abrar A Alzhrani, Mahmood Rasool, Sajjad Karim, Ahmed Alhejin, Absarul Haque, Mohamed Morsi, Mohamed Nabil Alama, Peter Natesan Pushparaj

**Affiliations:** 1 Department of Biological Sciences, Faculty of Science, King Abdulaziz University, Jeddah, Kingdom of Saudi Arabia; 2 Center of Excellence in Genomic Medicine Research, Faculty of Applied Medical Sciences, King Abdulaziz University, Jeddah, Kingdom of Saudi Arabia; 3 King Fahd Medical Research Center, Faculty of Applied Medical Sciences, King Abdulaziz University, Jeddah, Saudi Arabia; 4 Department of Cardiology, King Abdulaziz University Hospital, Jeddah, Saudi Arabia

**Keywords:** Fibrosis, Heart failure, cardiac remodeling, Extracellular matrix, Ferro ptosis

## Abstract

Cardiovascular disease (CVD) is one of the main causes of death in Saudi Arabia. Cardiac remodeling plays a critical role in the pathophysiology of heart failure. Major focus of our study was to identify crucial genes involved in the pathological remodeling of the heart caused by pressure overload. We utilized various in-silico tools to analyze and interpret microarray data obtained from the Gene Expression Omnibus (GEO) database (GSE120739), including GEO2R analysis, Metascape analysis, WebGestalt analysis, and IPA (Ingenuity pathway analysis). Our findings indicate that certain genes, including Cartilage Oligomeric Matrix Protein (COMP), collagen type VIII alpha 1 chain (COL8A1) and Lysyl Oxidase (LOX) under the influence caused by knockdown of KDM3A, were down regulated by the extracellular matrix pathway. Moreover, genes, such as Acyl-CoA Thioesterase 1 (ACOT1) were up regulated by the fatty acid metabolism pathway. Overexpression of lysine-specific demethylase 3A (KDM3A) leads to the up regulation of fibrosis-related genes COMP, COL8A1, and LOX and the down regulation of ACOT1, result in enhanced fibrosis and heart failure. Our results suggest that COMP, COL8A1, LOX, and ACOT1 warrant further investigation in the development of cardiac fibrosis and as potential biomarkers for causing heart failure.

## Background:

The most common cause of death worldwide is cardiovascular disease (CVD), which includes cardiac arrhythmia, heart failure, and myocardial infarction. In Saudi Arabia, more than 45% of all deaths are thought to be related to CVD. [1] Heart failure (HF) is a genetically complicated illness that involves numerous pathways and eventually results in a common phenotype of abnormal ventricular function and cardiac hypertrophy. Numerous studies have attempted to identify differentially expressed genes to discover biomarkers for disease prognosis and develop effective medications. [2] Myocardial fibrosis causes heart disorders and affects heart architecture. It also causes mechanical, electrical, and vasomotor dysfunction, all of which accelerate the progression of heart illness to heart failure. [3]. Cardiac fibroblasts (CFBs) are primary cells involved in the onset of myocardial fibrosis. By controlling the formation of the extracellular matrix (ECM), resident CFBs oversee the preservation of the structural integrity of the heart under homeostatic conditions. However, abnormal circumstances cause CFBs to activate, multiply, and release an excessive amount of ECM proteins, which helps form scar tissue. This scar substitutes for a healthy myocardium, causes the substrate to become arrhythmogenic, stiffens the heart, and results in adverse remodeling. [4]

Ferroptosis is a novel form of controlled cell death characterized by the accumulation of lipid peroxides, which damage the membrane structure. Lipid peroxidation involves the generation of reactive oxygen species (ROS) that damage lipids via carbon-carbon double bonds, such as polyunsaturated fatty acids (PUFAs) in cell membranes. [5] Reactive oxygen species (ROS) activation results in chamber remodeling and contractile dysfunction of the heart. [6] Ferroptosis contributes to cardiac fibrosis development. Several studies have investigated the relationship between ferroptosis and myocardial fibrosis. [7] lysine-specific demethylase 3A (KDM3A) is a well-known epigenetic activator that affects target gene transcription by removing the suppressive histone demethylation mark of histone H3 at lysine 9. Recent studies have shown that methylation is a crucial component of epigenetic machinery that is involved in heart hypertrophy, myocardial infarction, and other conditions. KDM3A plays a role in regulating the expression of extracellular matrix and fat metabolic genes during ferroptosis. [8] Therefore, it is of interest to examine the role of KDM3A in the regulation of the extracellular matrix and fat metabolic gene expression using next-generation knowledge discovery (NGKD) methods. Microarray data were obtained from the Gene Expression Omnibus (GEO) database (accession number GSE120739). It was demonstrated that KDM3A promotes left ventricular hypertrophy (LVH) by enhancing the expression of genes associated with fibrosis. KDM3A-overexpressing KDM3A-Tg and KDM3A knockout (KO) mice showed that KDM3A is required to enhance LVH in response to pressure overload induced by transverse aortic constriction (TAC). Thus, over-representation analysis (ORA) and gene set enrichment analysis (GSEA) indicated that KDM3A controls extracellular matrix biology and metabolism pathways, triggering fibrosis and enhancing ECM gene expression.

## Methods:

## Data source:

Gene expression microarray data from the four left ventricles of mouse heart samples were collected from the Gene Expression Omnibus (GEO) database (accession number GSE120739). The study was designed in silico using statistical web-based tools to analyze genetic data. We analyzed GEO data using NGKD tools.

## GEO2R analysis:

We used GEO2R analysis with Benjamin and Hochberg (false discovery rate), and the significance level cut-off was 0.05, with UMAP, boxplot graph, and mean difference plot from the National Center for Biotechnology Information (NCBI). In the GEO2R analysis, NCBI was generated to identify differentially expressed genes in four samples and to compare two groups: the test group with KDM3A mice (KDM3A-overexpressing) as the control group.

## Metascape analysis:

The analysis was performed to annotate the gene list. In Metascape, we analyzed the gene list with the parameters of input and analysis as species: M. musculus (145) and the input of the gene symbol from the gene list, with the logFC cut of the gene list between (-2 and 2) and a P-value less than 0.05.

## WebGestalt analysis:

In WebGestalt, the parameters we used for ORA analysis (over-representation analysis), the organism of interest is Mus musculus; we used only the gene symbol of the gene list of the logFC between (-2 and 2) and P-value less than 0.05, For GSEA analysis (gene set enrichment analysis) to analyze the gene expression profiles. The organism of interest is Mus musculus, we used the gene symbol of the gene list of the logFC between (-2 and 2), the P-value less than 0.05, and the logFC value as a parameter.

## Ingenuity Pathway Analysis:

In the ingenuity pathway analysis (IPA) with statistical parameters of the logFC cut of the gene list between (-2 and 2) and P-value less than 0.05 to obtain differentially expressed gene for further downstream analysis.

## Results:

**Figure 1:**
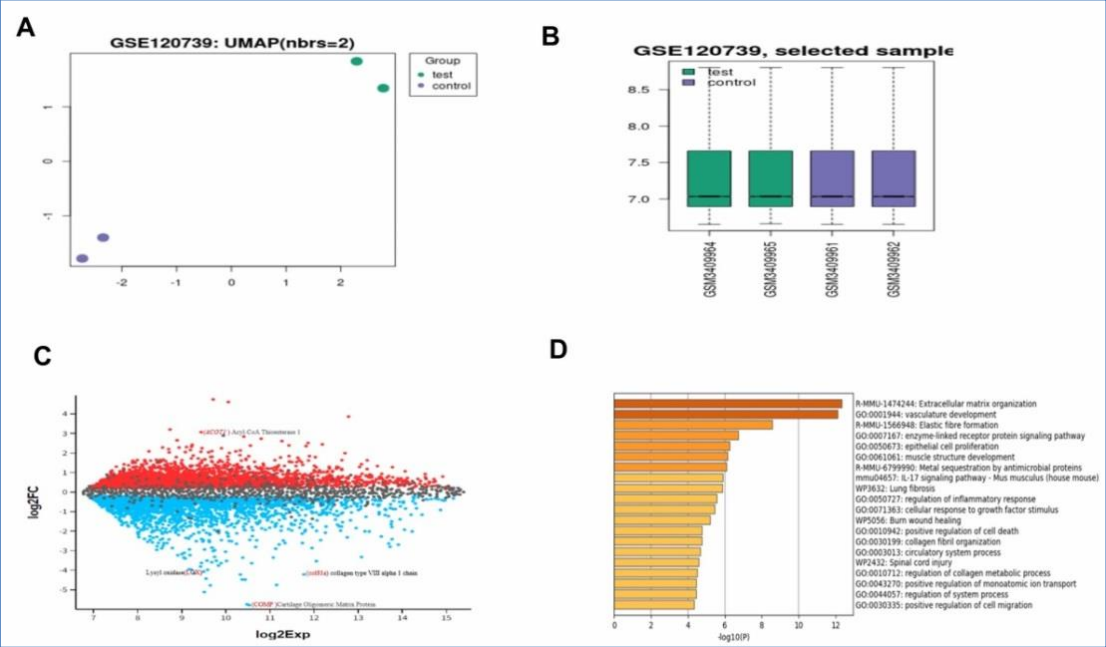
A) UMAP graph from the GEO2R analysis B) Box plot shows the normalization of the selected samples in the microarray dataset C) Mean difference plot graph from the GEO2R analysis showing significant up regulation and down regulation of genes. D) A bar graph from the Metascape analysis with the top 20 clusters, the "extracellular matrix organization" is the most significant.

From the GEO2R analysis, A UMAP graph shows four samples from the left ventricle of the heart; the top green circles are the test group, and the down purple circles represent the control group (Figure 1A). A boxplot graph showing the distribution of the values of the selected sample (Figure 1B) and the mean difference plot graph from the GEO2R analysis shows significant up regulation and down regulation of genes (Figure 1C). The Metascape analysis with the use of the pathway and process enrichment analysis revealed the top 20 clusters of the enriched terms from the gene list, on the top "Extracellular matrix organization" (R-MMU-1474244) with a log 10(P) of -12.50, (vasculature development) (GO: 0001944) with a log of 10(p) of -12.16, both of which are related to our findings (Figure 1D). We performed an ORA analysis with the functional database as gene ontology.

## Biological process:

**Figure 2:**
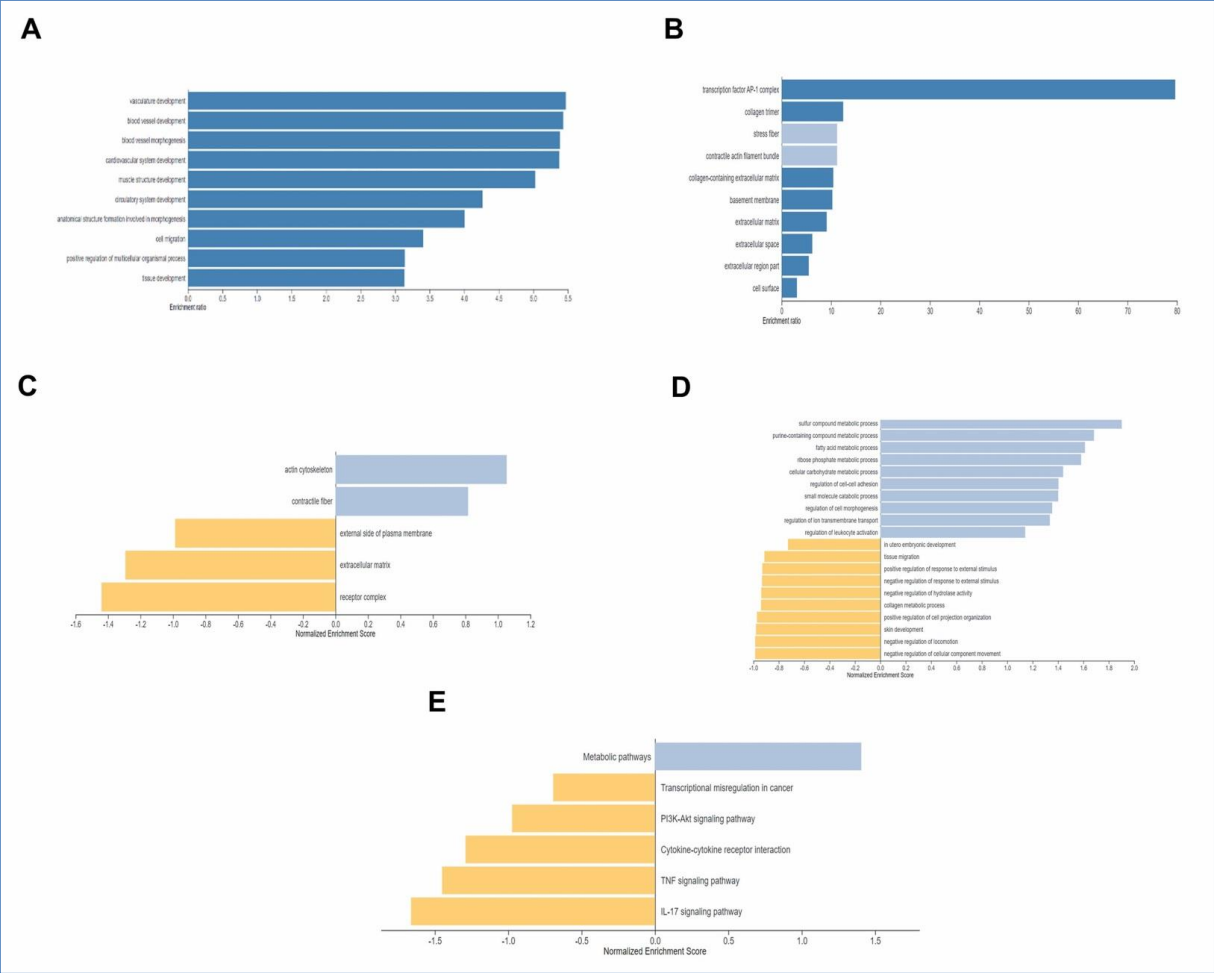
A) Bar graph of ORA analysis with gene ontology biological process with enriched gene set (COMP, LOX, and COL81A) from WebGestalt; B) Bar graph of ORA analysis with gene ontology Cellular Component with a significant result, the enriched gene set has the relative genes (COMP, LOX, and COL8A1) from WebGestalt; C) Bar graph of GSEA analysis with Cellular Component no Redundant with down regulated colored in yellow has COMP LOX, and COL8A1 from WebGestalt; D) Bar graph of GSEA analysis with gene ontology biological Process no Redundant, colored in light blue has ACOT1, from WebGestalt; E) Bar graph of GSEA analysis with pathway EGG, with the up regulation colored in light blue of COMP and down regulation colored in yellow of ACOT1, from WebGestalt.

We found Cartilage Oligomeric Matrix Protein (COMP), collagen type VIII alpha 1 chain (COL8A1), and Lysyl Oxidase (LOX) in (vasculature development) (GO: 0001944) with FDR 3.7790e-7. All three genes were identified in (blood vessel development) (GO: 0001568) with FDR 4.4790e-7 (blood vessel morphogenesis) (GO: 0048514) with FDR 0.0000059751 and (cardiovascular system development) (GO: 0072358) with FDR 3.7790e-7. We also find COMP in "tissue development "(GO: 0009888) with FDR 0.0000030210 (Figure 2A).

Functional databases, such as Gene Ontology Cellular Components, We found COMP, LOX, and COL8A1 gene in "collagen- containing extracellular matrix "(GO: 662623) with FDR <2.2e-16," extracellular matrix" (GO: 0031012) with FDR <2.2e-16," extracellular space" (GO: 0005615) and "extracellular region part" with FDR <2.2e-16. We also found LOX and COL8A1 in the "collagen timer" (GO: 0005581) with an FDR of 0.011753 (Figure 2B). GSEA analysis with Gene Ontology Cellular Component_noRedundant revealed no significantly down regulated gene set with (FDR >0.05) or "extracellular matrix" (GO: 0031012) with FDR (0.24688). The gene set had COMP with a score of -5.7, LOX with a score of -4.06, and COL8A1 with a score of -3.5 (Figure 2C). In another analysis of pathway gene ontology biological process with no redundancy, we found no statistically significant upregulated gene set with (FDR > 0.05). "Fatty acid metabolic processes" (GO: 0006631) with a false discovery rate (FDR) of 0. 35104). the gene set ACOT1 had a score of 3.06 (Figure 2D). In another GSEA with KEGG pathway analysis, we did not find any significantly upregulated or downregulated gene sets with (FDR > 0.05). An unregulated gene set, "Metabolic pathways" (GO: mmu01100), with FDR (0.10529), had an Acyl-CoA Thioesterase 1 (ACOT1) gene score of 3.06. A down regulated gene set" PI3K-Akt signaling pathway" (GO: mmu04151) with FDR 0.60665, this gene set has a COMP gene with a score of -5.7 (Figure 2E).

In WebGestalt, a significantly down regulated gene set is shown for the (extracellular matrix) and an upregulated gene set is shown for (the metabolic pathway). Under ORA analysis, the genes COMP, COL8A1, and LOX were shown in the (vasculature development) gene set. All three genes were also found to be involved in (cardiovascular system development), (collagen-containing extracellular matrix), (extracellular matrix), (extracellular space) and (extracellular region part), all of which were significant in the ORA analysis (Figure 2A and Figure 2B). In the GSEA, all three genes were found in the downregulated gene set (extracellular matrix). The ACOT1 gene was upregulated in the gene set (fatty acid metabolic process) and (metabolic pathways) (Figure 2C-Figure 2E). From IPA analysis, we identified 329 Ingenuity Canonical Pathways and found different pathways related to heart disease and our genes overlapping with these pathways, such as the Apelin Cardiac Fibroblast Signaling Pathway, Dilated Cardiomyopathy Signaling Pathway, Ferroptosis Signaling Pathway, Cardiac Hypertrophy Signaling (Enhanced), Inhibition of Matrix Metalloproteases, PI3K/AKT Signaling, p53 Signaling, and VEGF Signaling and Regulation of the Epithelial-Mesenchymal Transition Pathway (Table 1).

**Figure 3:**
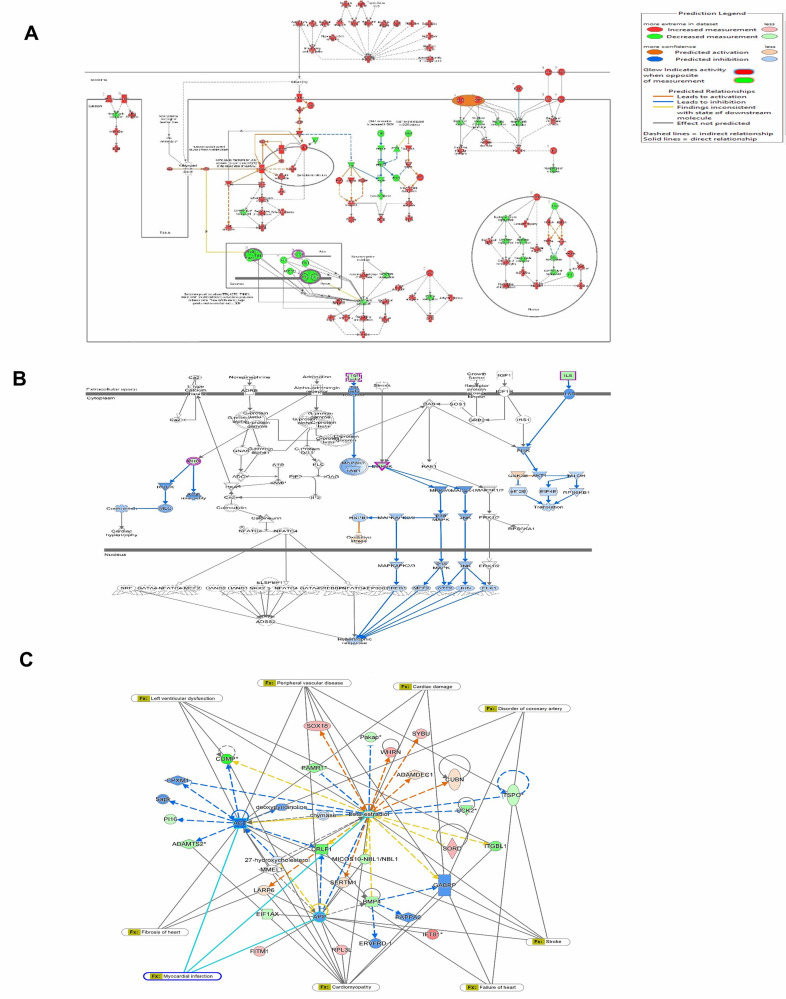
Differentially regulated pathways obtained from IPA analysis A. Dilated Cardiomyopathy Signaling Pathway B. Cardiac Hypertrophy Signaling Pathway. C. Gene Network with disease overlay of cardiovascular disease from IPA analysis.

**Figure 4:**
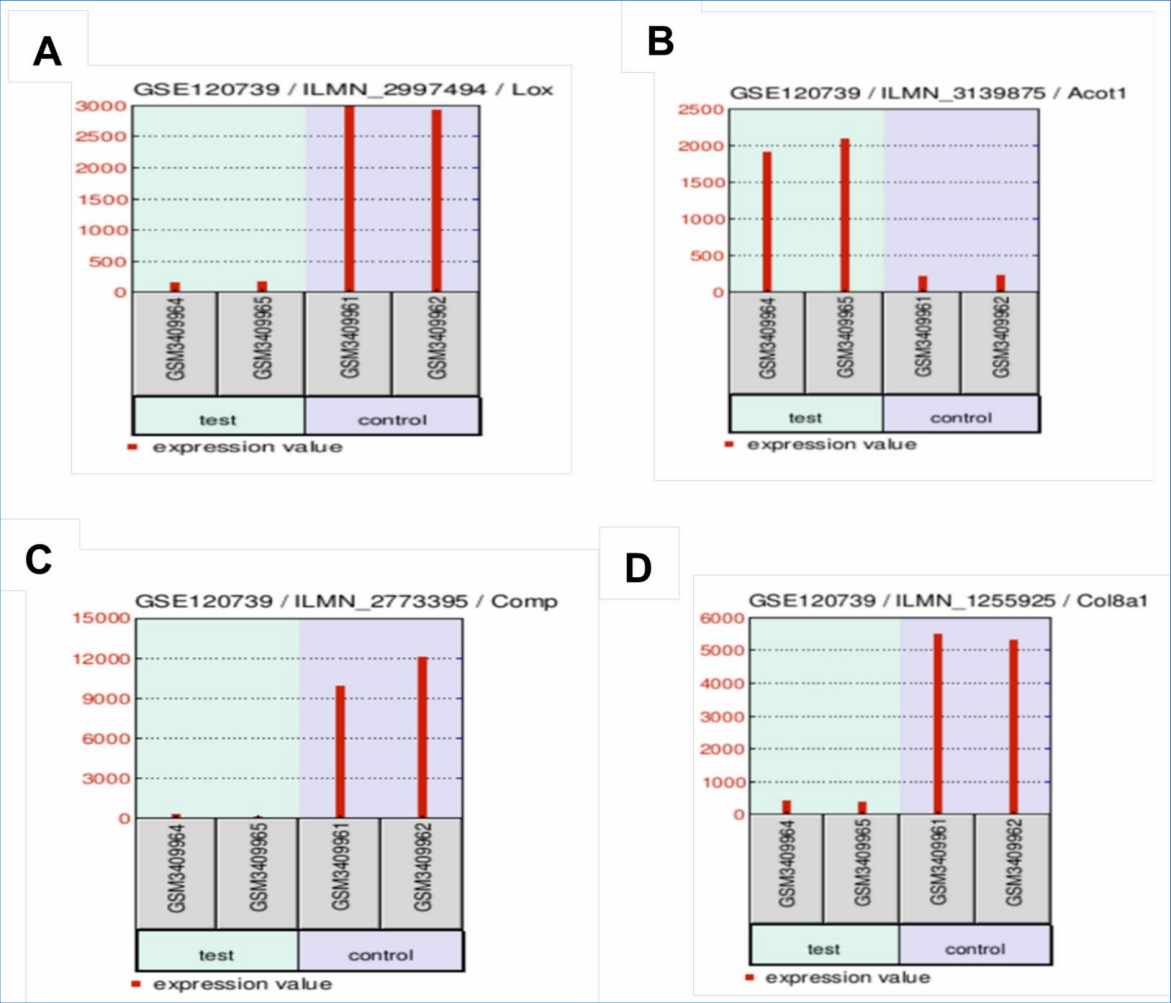
Results of gene expression form the GEO2R analysis; A. The figure shows the down regulation of LOX gene; B. up regulation of The ACOT1 gene; C. down regulation of COMP gene and D the down regulation of COL8A1gene and its sample values.

We identified several signaling pathways associated with cardiac remodeling, such as the dilated cardiomyopathy signaling pathway, with an a-log (p-value) of 1.44, and a ratio of 0.02, and a Z-score of 0. Most of the genes were upregulated in this pathway (Figure 3A). We also observed Cardiac Hypertrophy Signaling, which is related to our findings, with an a-log (p-value) of 0.909, ratio of 0.00923, and Z score of -2. Most genes were down regulated in this pathway (Figure 3B). For the p53 signaling pathway, the -log (p-value) was 0.421, the ratio was 0.0102, and the Z score was 0. Most genes in this pathway were downregulated, and in the regulation of Epithelial Mesenchymal Transition, the -log(p-value) was 0.613, the ratio was 0.0103, Z score was 0. We found that most genes in this pathway were down regulated. We obtained a gene network disease overlay for cardiovascular illness from IPA analysis, which showed several genes linked to several heart diseases and cardiovascular disorders, and one of our resultant genes, the COMP gene (Figure 3C). We discovered that some genes that are affected by the knockdown of KDM3A, such as COMP, COL8A1, and LOX, are down regulated in the extracellular matrix pathway, and some of the genes such as ACOT1 are upregulated in the underlying fatty acid metabolism pathway (Figure 4). From our results, we observed the differential regulation of COMP, LOX, COL8A1 and ACOT1 genes in heart failure and their roles in promoting fibrosis in two pathways: the extracellular matrix and the metabolic pathway. We found in our analysis that the four genes COMP (Cartilage Oligomeric Matrix Protein), LOX (Lysyl Oxidase), COL8A1 (collagen type VIII alpha 1 chain) and ACOT1 (Acyl-CoA Thioesterase 1) are fibrosis-enhancing genes that promote heart failure.

## Discussion:

Cardiac remodeling is a crucial factor in the pathophysiology of heart failure according to ongoing research in recent years. To shed new light on myocardial fibrosis, the current study identified critical genes involved in cardiac remodeling. [9]

Adverse extracellular matrix (ECM) remodeling, a defining feature of heart failure (HF), is controlled by collagen cross-linking enzyme lysyl oxidase (LOX). Some studies have investigated the effectiveness of LOX inhibition in preventing adverse left ventricular (LV) remodeling and dysfunction in an experimental mouse model of HF. [10] In our analysis, the LOX gene was downregulated by GSEA, which underlines the extracellular matrix pathway.

LOX promotes the development and deposition of stiff collagen fibers. Consequently, cardiac fibrosis increases collagen deposition and stiffness. Despite the fibrotic response's goal of maintaining tissue integrity, pathological fibrosis gradually impairs LV function, which is associated with poor prognosis and a significantly increased risk of HF. [11] In a mouse model of TAC (KDM3A knockdown), LOX expression was down regulated, indicating that overexpression of KDM3A induces fibrosis-related genes. A previous study showed that mice lacking colVIIIa1 (COL8A1) exhibited reduced myofibroblast differentiation, fibrosis, and LV dilatation in response to pressure overload. [12] Another study showed that mice lacking COMP had reduced fibrosis owing to abnormalities in collagen secretion. [13] Both genes were down regulated in the GSEA analysis, underlining the extracellular matrix pathway in our analysis.

In our analysis, the expressions of COMP and col8a1 were down regulated in a mouse model of TAC (KDM3A knockdown). COMP and col8a1 are expressed in cardiac ECM. According to various studies, both genes have been shown to play a role in fibrosis. In fibrosis, excess matrix stiffens the ECM, leading to cellular dysfunction and heart failure. [14] [15] KDM3A knockdown results in the down regulation of COMP and col8a1which reduce fibrosis and improves cardiac function, indicating the presence of genetic modifiers of KDM3A-regulated hypertrophic remodeling. [16] Down regulation of these genes inhibits cardiac fibrosis, suggesting that their overexpression results in fibrosis.

A previous study showed that the overexpression of Acot1 prevents cardiac dysfunction by reducing oxidative stress. Acot1 may protect cardiomyocytes against ferroptosis, whereas Acot1 knockdown makes cardiomyocytes more susceptible to ferroptosis. [7] In our analysis, we observed that ACOT1 was upregulated, thereby underlining the fatty acid metabolic pathway. In our data analysis, we noticed that the knockdown of KDM3A resulted in the upregulation of the ACOT1 gene, which plays a role in ferroptosis. Ferroptosis is involved in myocardial fibrosis and several studies have investigated the relationship between ferroptosis and myocardial fibrosis. [17] Acot1 knockdown increases oxidative stress and inflammation and most likely results in fibrosis. [7] Researchers have proposed that LOX may play a role in a small proportion of cases of ferroptosis. However, it remains unclear whether these enzymatic processes can cause ferroptosis. [18] One study has suggested that treating cardiomyopathy by inhibiting ferroptosis could help prevent heart failure. Further research is required to examine the clinical effects of this therapeutic approach. [19] We used ingenuity pathway analysis (IPA), to interpret biological functions and signaling pathways, Our IPA-based canonical pathway analysis identified several signaling pathways associated with cardiac remodeling.

We revealed a dilated cardiomyopathy signaling pathway, which is characterized by normal left ventricular wall thickness with ventricular chamber expansion and systolic dysfunction. [20] Focal myocardial fibrosis is seen in one-third to two-thirds (30-66%) of DCM patients, according to several investigations that used LGE-CMR. Cardiac fibrosis and disease severity are correlated with DCM. [21] According to a previous study, generalized myocyte death throughout the ventricular wall causes dilated cardiomyopathy to manifest. Caspase activation is an effector pathway that is associated with myocyte apoptosis. [22] Previous research on ischemic heart disorders has demonstrated that changes in the Akt signaling pathway promote apoptosis. [23] Similar to ventricular hypertrophy, left ventricular chamber dilatation is linked to myocyte loss in the heart, which causes fibrosis in the focal areas of fibrosis. [24]

Further studies have shown that AKT activation prevents cardiomyocyte death, resulting in cardiac dysfunction. [25] In our analysis of the dilated cardiomyopathy signaling pathway, we found that AKT, which plays a role in the activation of cardiomyocyte apoptosis, was down regulated. Activation of Cardiomyocyte apoptosis results in fibrosis, DCM, and heart failure.

We also observed cardiac hypertrophy Signaling, which is related to our findings, and the role of cardiac fibroblasts in the hypertrophic heart is crucial for cardiac remodeling. Heart fibroblasts release a variety of growth factors and extracellular matrix elements that cause and alter cardiomyocyte hypertrophy. [26] Cardiovascular disorders are significantly affected by the RhoA-Rho kinase (ROCK) signaling pathway. [27] Owing to its effect on fibroblasts, ROCK plays a significant role in the regulation of cardiovascular fibrosis. ROCK may mediate fibrosis and stiffening of the underlying vasculature, heart failure, cardiac arrhythmias, and recovery from myocardial infarction. A previous study showed that ROCK2 deletion in cardiac fibroblasts leads to decreased cardiac hypertrophy. [28] in cardiac hypertrophy signaling, we found that the down regulation of ROCK results in cardiac hypertrophy. We found that the down regulation of different genes, such as CREB1, MEF2, ATF2, JUN, and ELK1, resulted in a hypertrophic response.

We also identified p53 signaling, which plays a role in cardiac fibrosis, by IPA analysis. A study showed a mechanism controlling the amount and timing of fibrosis in left ventricular pressure overload by controlling cardiac fibroblast accumulation and extracellular matrix secretion, which is partially mediated by p53-dependent cell cycle control. [29] In a mouse model of TAC, serpinE2 knockdown reduced cardiac fibrosis. [30] The expression of two genes (CCNG1 and SERPINE2) was previously reported to be regulated by p53. [31] In our analysis, we found that serpinE2 and CCNG1 were down regulated, as were other genes, such as TP53, DRAM1, and TIGAR in the p53 signaling pathway.

Another pathway related to our findings is the regulation of epithelial-mesenchymal transition (EMT). EMT is a biological and molecular process by which cells lose their identity as epithelial cells, characterized by stable intercellular connections and apical-basal polarity, and develop a mesenchymal phenotype, including a fibroblast-like gene expression profile, cytoskeletal and morphological rearrangement, the ability to migrate, and the production of extracellular matrix (ECM). [32]

Upon TGF-β stimulation, endothelial cells give rise to fibroblasts. Endothelial cells undergo Endothelial Mesenchymal Transition (EndoMT), which initiates the development of cardiac fibrosis. As TGF-β-driven EMT is also responsible for the production of cardiac fibroblasts throughout development, the EMT process may be implicated in cardiac fibrosis in addition to EndoMT [33]. A previous study showed that LOX plays a role in the regulation of EMT-inducing transcription factors such as SNAI1 and SNAI2. These proteins are involved in the EMT during embryonic development. SNAI1 mRNA levels were not significantly changed by LOX knockdown; however, the SNAI2 mRNA levels were significantly decreased. [34] In our analysis, we found that the LOX gene was knocked down and most other gene, such as SNAI1 and SNAI2, were down regulated.

## Conclusion:

In conclusion, the current study utilized NGKD tools to identify myocardial fibrosis-related genes in the hearts of mice with transaortic constriction, either with a knockout of KDM3A or overexpression of KDM3A. These findings indicate that increased expression of KDM3A leads to the up regulation of the fibrosis-related genes COMP, COL8A1, and LOX, as well as the down regulation of ACOT1, all of which contribute to the development of heart failure through enhanced fibrosis. Knockdown of KDM3A inhibits the activation and differentiation of cardiac fibroblasts into myofibroblasts, thereby preventing cardiac fibrosis. Furthermore, our results suggest a connection between ferroptosis and myocardial fibrosis and that ACOT1 is involved in ferroptosis, which leads to the initiation of oxidized lipid metabolism and generation of reactive oxygen species (ROS), ultimately resulting in biological membrane damage and fibrosis. Therefore, COMP, COL8A1, LOX, and ACOT1 are potential targets for future investigations as contributors to cardiac fibrosis and biomarkers for heart failure.

## Future recommendations:

In the future, more studies using animal models are needed to investigate and validate the genes as well as the pathways related to these genes implicated in cardiac fibrosis to uncover potential biomarkers for cardiac failure.

## Figures and Tables

**Table 1:** Differentially regulated canonical pathways related to heart diseases

**Ingenuity Canonical Pathways**	**-log(p-value)**	**Ratio**	**z-score**
Apelin Cardiac Fibroblast Signaling Pathway	2.26	0.087	0
Dilated Cardiomyopathy Signaling Pathway	1.44	0.02	0
Cardiac Hypertrophy Signaling (Enhanced)	0.909	0.00923	-2
Ferroptosis Signaling Pathway	0.872	0.0152	0
Inhibition of Matrix Metalloproteases	0.763	0.0256	0
P13K/AKT Signaling	0.597	0.01	0
p53 Signaling	0.421	0.0102	0
VEGF Signaling	0.418	0.0101	0
Regulation of the Epithelial- Mesenchymal Transition Pathway	0.613	0.0103	0
